# Prospective evaluation of fexapotide triflutate injection treatment of Grade Group 1 prostate cancer: 4-year results

**DOI:** 10.1007/s00345-020-03127-w

**Published:** 2020-02-22

**Authors:** Neal Shore, Steven A. Kaplan, Ronald Tutrone, Richard Levin, James Bailen, Alan Hay, Susan Kalota, Mohamed Bidair, Sheldon Freedman, Kenneth Goldberg, Frederick Snoy, Jonathan I. Epstein

**Affiliations:** 1 Carolina Urologic Research Center, Myrtle Beach, SC USA; 2 Icahn School of Medicine at Mount Sinai, New York, NY USA; 3 Chesapeake Urology Research Associates, Baltimore, MD USA; 4 Chesapeake Urology Research Associates, Towson, MD USA; 5 First Urology, Louisville, KY USA; 6 Willamette Urology, Salem, OR USA; 7 Urological Associates of Southern Arizona, Tucson, AZ USA; 8 San Diego Clinical Trials, San Diego, CA USA; 9 Freedman Urology, Las Vegas, NV USA; 10 UT Southwestern Department of Urology, Lewisville, TX USA; 11 Urology Group of New Mexico, Albuquerque, NM USA; 12 Johns Hopkins Medical Institutions, Baltimore, MD USA

**Keywords:** Prostate cancer, Fexapotide triflutate, Urology, Focal therapy

## Abstract

**Purpose:**

This study was undertaken to determine the safety and efficacy of fexapotide triflutate (FT) 2.5 mg and 15 mg for the treatment of Grade Group 1 prostate cancer.

**Methods:**

Prospective randomized transrectal intraprostatic single injection FT 2.5 mg (*n* = 49), FT 15 mg (*n* = 48) and control active surveillance (AS) (*n* = 49) groups were compared in 146 patients at 28 U.S. sites, with elective AS crossover (*n* = 18) to FT after first follow-up biopsy at 45 days. Patients were followed for 5 years including biopsies (baseline, 45 days, and 18, 36, and 54 months thereafter), and urological evaluations with PSA every 6 months. Patients with Gleason grade increase or who elected surgical or radiotherapeutic intervention exited the study and were cumulatively included in the data analysis. Percentage of normal biopsies in baseline focus quadrant, tumor grades, and volumes; and outcomes including Gleason grade in entire prostate as well as treated prostate lobe, interventions associated with Gleason grade increase and total incidence of interventions were assessed.

**Results:**

Significantly improved long-term clinical outcomes were found after 4-year follow-up, with percentages of patients progressing to interventions with and without Gleason grade increase significantly reduced by FT single treatment. Results in the FT 15-mg group were superior to the FT 2.5-mg dose group. There were no drug-related serious adverse events (SAEs).

**Conclusions:**

FT showed statistically significant long-term efficacy in the treatment of Grade Group 1 patients regarding clinical and pathological progression. FT 15 mg showed superior results to FT 2.5 mg. There were no drug-related SAEs; FT injection was well tolerated.

## Introduction

There is an unmet need for treatments for low-grade prostate cancer (PCa) that produce minimal collateral tissue damage and unintended sexual, urinary, and bowel function side effects [1–23]. Grade groups were first proposed by authors at Johns Hopkins Hospital led by Dr. Epstein [21], validated in a large multi-institutional study [22], and subsequently endorsed by the 2014 International Society of Urological Pathology Consensus Conference [23] and the WHO. Gleason Grade Group 1 is the most commonly diagnosed PCa and is considered very low-to-low risk by the National Comprehensive Cancer Network (NCCN) criteria. However, these cancers, albeit low risk, are still infrequently capable of biologic progression, and thus cause ongoing patient anxiety. Often, these low-risk cancer patients may still receive interventional therapies which can result in urinary, bowel, and sexual side effects. The natural history of these indolent, well-differentiated prostate cancers, and their management can be influenced by age, performance status, co-morbidities, sociodemographic factors, and genomic factors [24–36]. An overarching difficulty in the management of low-risk prostate cancer patients is that validations with long-term prospective outcomes are essentially prohibitively delayed due to the 15–20 years required for mortality data and the unrealistic likelihood of adequate recruitment. Most authorities, therefore, agree that more pragmatic parameters of objective clinical and pathological progression are currently the most realistic approach to assessment of efficacy and safety [1–20].

Fexapotide triflutate (FT) is a new molecular entity with pro-apoptotic effects, delivered by intraprostatic injection, which has been investigated for the treatment of both lower urinary tract symptoms due to BPH and for low-grade localized PCa. FT has been administered in prospective randomized placebo controlled double-blinded long-term BPH trials involving over 1200 men, and shown to be well tolerated and to provide long-term BPH efficacy without significant adverse effects [37–40]. This report presents 4-year data from a 7-year study of 146 patients with Grade Group 1 (Gleason 6) T1c PCa, randomized to treatment with Fexapotide Triflutate (FT) or active surveillance (AS), and which included an elective crossover (CO) group from AS to active drug. To our knowledge, this is the first long-term prospective randomized-controlled study of an intraprostatic molecular injectable treatment for low-grade localized PCa to be reported.

## Patients and methods

Study NX03-0040 was a Phase 2 multi-center prospective open label two-dose (2.5 mg and 15 mg) level clinical safety and efficacy evaluation of FT injection for the treatment of low-risk, localized (T1c) PCa, comparing FT 2.5-mg and 15-mg single dose to AS, and including an elective AS CO group. The study was conducted at 28 U.S. urological investigational sites (44 sites approved and initiated; 30 with patient screening; 28 with patient enrollments) from 2012 to 2018, with protocols approved by institutional review boards (clinicaltrials.gov identifier NCT01620515). Informed consent was obtained from all individual participants included in the study. Patients were enrolled based on the following criteria: diagnosis of T1c PCa Grade Group 1; prostate biopsy within previous 6 months (≥ 10 cores; single core positive; ≤ 50% in the single positive core); PSA ≤ 10 ng/mL; and no previous treatments for prostate cancer. Patients’ assessments additionally included International Prostate Symptom Score (IPSS) at baseline and at follow-up. Patients were centrally randomized in a 1:1:1 ratio of FT 2.5-mg:FT 15-mg:AS; by a computer-generated randomization schedule executed by non-study personnel at an independent randomization service provider with no contact except by interactive voice response system. Patients and investigational staff were blinded as to which FT dosages (FT 15 mg vs 2.5 mg) were administered in the treatment groups. AS patients were not given sham treatments. 267 patients were screened, with 146 patients enrolled, and 141 patients qualified for efficacy analysis (Intent-to-Treat). Three groups were initially randomized (Study Population, Table 1): FT 15 mg (*n* = 48 randomized, *n* = 47 injected), FT 2.5 mg (*n* = 49 randomized, *n* = 48 injected) by transrectal intraprostatic injection; and AS (*n* = 49), and two smaller elective groups of AS to FT COs after the 6-week post-randomization biopsy (FT 2.5 mg *n* = 10; FT 15 mg *n* = 8). CO patients were required to have completed the initial 6 weeks per protocol and to continue to fulfill inclusion/exclusion criteria at the time of CO. Seven initial visits included: Visit 1: screening; Visit 2 (Day 1): dosing; Visit 3 (Day 2); Visit 4 (Day 4); Visit 5 (Day 10); Visit 6 (Day 45): biopsy; and Visit 7 (Day 60); biopsy follow-up. Follow-up Visits (up to 5 years): PSA (every 6 months); physical examination (every 12 months); prostate biopsies (every 18 months). Blinded prostate biopsies at screening and at 45 days were read by Dr. J. Epstein, Johns Hopkins Medical Institutions. Long-term biopsies after 45 days were reported by the local pathology services in conjunction with the individual investigational sites. Interim safety analyses by an Independent Safety Data Monitoring Committee (IDMC) were scheduled (i) after the first ten patients dosed with FT (both dosage groups combined *n* = 10), (ii) after the first ten FT 15-mg dose group, and (iii) after *n* = 50 both groups FT combined. If there was excessive drug-related toxicity, the IDMC would stop the trial. The injection procedure for FT intraprostatic treatment has been described in detail previously [37–40] and was directed to the same quadrant as the cancer on initial biopsy. FT is supplied as a sterile lyophilized powder that is reconstituted in 10-mL sterile phosphate buffered saline and injected into prostate by the transrectal route under transrectal ultrasound (TRUS) guidance by standard technique using a conventional #22 gauge sterile needle.

**Table 1:** Study population

Group	FT 2.5 mg	FT 15 mg	AS^a^
*N* randomized	49	48	49
Age (mean in years) (SD)	64.0 (7.1)	64.4 (7.7)	62.6 (7.0)
Race			
Caucasian	47	45	43
African-American	2	2	4
Other	0	1^b^	2^c^
PSA (mean) (SD)	4.7 (2.3)	4.2 (1.9)	4.3 (1.9)
IPSS (mean) (SD)	9.6 (6.4)	8.3 (5.8)	10.9 (8.8)
Prostate volume (mean) (SD)	52.3 (23.1)	46.1 (15.9)	49.7 (24.7)
Clinical stage t(1c) t(2a)	49/49	48/48	49/49
Biopsy (ISUP Grade 1)	49/49	48/48	49/49
Gleason 3 + 3 number of positive cores			
1	49/49	48/48	49/49
Mean lesion % of positive core (SD)	9.5% (8.8%)	12.3% (12.2%)	10.4% (11.5%)

^a^CO patients are included in AS at baseline as randomized. CO patients are included in their respective FT groups for outcome listings in Table 3

^b^Caucasian–American Indian

^c^American–Indian; Asian

### Statistical methods

#### Statistical analysis plan

Primary endpoint was presence or absence of cancer in initially positive baseline quadrant focus (BLF) based on blinded assessments of prostate biopsies and PSA. Secondary endpoints included: change in median tumor grade in each treatment group in (i) BLF, and (ii) in the entire prostate; and change in mean tumor volume in each treatment group (estimated by tumor % in biopsy section) in (iii) BLF, and (iv) in the entire prostate. Cancer progression was assessed by clinical evaluation, long-term serial biopsies (every 18 months or earlier, if for cause), PSA (every 6 months or earlier), incidences of surgery or radiotherapy for PCa with or without histological upgrades, and surgical pathology results. All patients with clinical and/or pathological progression (increased Gleason grade > 3 + 3 in their prostate overall, or post-randomization treatment with prostatectomy or radiotherapy and/or chemotherapy) were included in the statistical analysis regardless of when they exited the study. CO subjects were included in the AS group for the initial 6-week protocol measures only. After CO, they were included in the FT group of their respective FT dosage received. Subjects lost to follow-up or who dropped out, without clinical or pathological progression, were included in pathological analysis at 18, 36, and 48 months if there was ≥ 18, ≥ 36, or ≥ 48-month biopsy data, respectively, but were excluded if they were lost prior to 18-month biopsy, and were excluded from later calculations if subsequently lost or withdrawn. Corrections for multiple comparisons were not done for the significance values which are reported separately. For BLF in patients with surgery or radiation and no biopsy, baseline was carried forward. Statistical assistance was provided by Sherryl Baker PhD (Everest Clinical Research, Little Falls NJ).

## Results

**Fig. 1:**
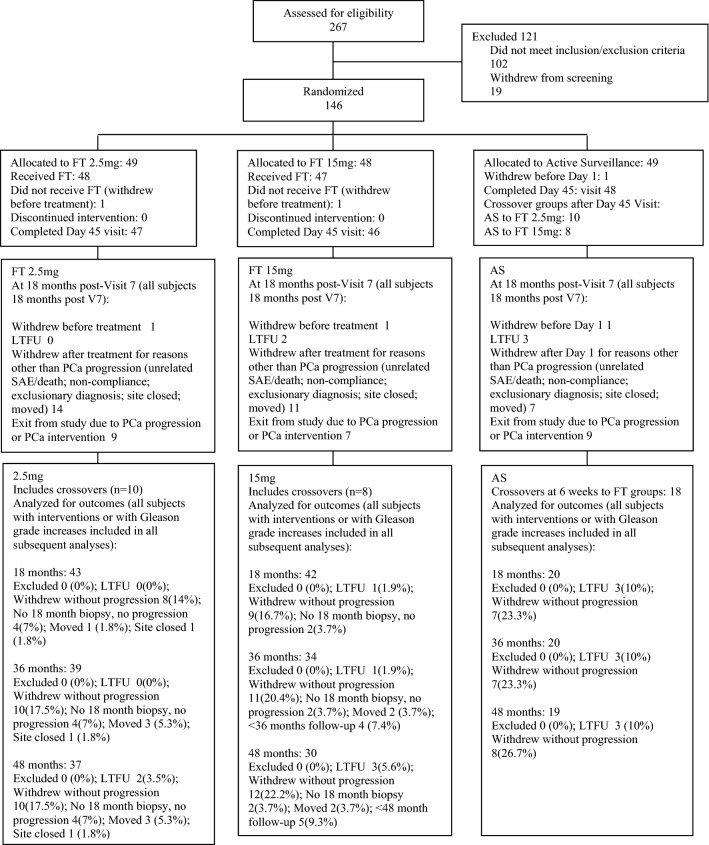
Consort diagram

Patient disposition is summarized in CONSORT diagram (Fig. 1). First patient enrollment was June 6, 2012, and last patient enrollment (*n* = 146) was Feb 11, 2014. Long-term follow-up data were analyzed as of 78-month post-first patient enrollment (which was 5-year post-last patient enrollment). Mean and (median) ages at randomization were AS 63.2 (64), FT 2.5 mg 63.7 (63), and FT 15 mg 63.9 (65) years. There were two (1.37%) patients who dropped out before (day 1) study treatment and two (1.37%) dropped out (without cause) before the first post-randomization biopsy at 45 days. Prior to the 18-month biopsy assessment *n* = 14 (9.6%) of subjects had exited the study due to pathological progression of their PCa and *n* = 11 (7.5%) exited without progression due to the decision to receive interventions such as surgery or radiotherapy for their PCa, with 5 (3.4%) patients lost to follow-up (LTFU) and 32 (21%) withdrew consent without documented progression of their PCa.

### Safety results

Consistent with previous U.S. clinical trial data (*n* > 1200) for FT 2.5 mg, the vast majority of drug injection-related adverse events (AEs) included injection procedure-related AEs (e.g., transient hematuria in 6.1%; transient dysuria in 1.7%; transient hematospermia in 2.6%) antibiotic prophylaxis-related AEs (e.g., transient diarrhea in 26.1%; transient nausea in 8.7%; transient constipation in 1.7%) (Table 2), and resolved uneventfully. There were no FT drug-related AEs. This is consistent with clinical pharmacokinetic (PK) data which have shown no detectable levels in plasma at 1-, 5-, 10-, and 20-min post-injection, and with clinical immunological data showing no antibody response to FT after injection and after re-injection [37–40]. In the present study, total testosterone levels in FT groups were not reduced compared to controls at 45 days. There were no long-term clinically significant lower urinary tract side effects in any subjects. Lower urinary tract symptoms (LUTS) changes as measured by IPSS after 6 weeks showed no difference between pooled FT subjects and AS [mean change from baseline all FT-treated subjects − 1.05 (SD 3.83) vs AS 0.02 (3.41), NS, *t* test].

**Table 2:** Treatment-related adverse events (AEs) in the first year after treatment

AE	Combined FT (*n* = 115) group AEs: *n* (%)	FT 2.5 mg (*n* = 59) group AEs: *n* (%)	FT 15 mg (*n* = 56) group AEs: *n* (%)	AS^a^ (*n* = 49) group AEs: *n* (%)
Procedure-related^b^				
Dysuria	2 (1.7)	1 (1.7)	1 (1.8)	0
Haematochezia	3 (2.6)	0	3 (5.4)	0
Haematospermia	3 (2.6)	1 (1.7)	2 (3.6)	0
Haematuria	7 (6.1)	3 (5.1)	4 (7.1)	0
Penile pain	2 (1.7)	0	2 (3.6)	0
Rectal pain	0	0	0	1 (2.0)
Antibiotic-related^b^				
Arthralgia	2 (1.7)	2 (3.4)	0	0
Constipation	2 (1.7)	0	2 (3.6)	0
Diarrhoea	30 (26.1)	19 (32.2)	11 (19.6)	1 (2.0)
Dysgeusia	3 (2.6)	0	3 (5.4)	0
Headache	3 (2.6)	3 (5.1)	0	0
Nausea	10 (8.7)	6 (10.2)	4 (7.1)	0

All FT-related AEs with combined FT groups *n* > 1, and all related AS AEs ≥ 1%. Excludes biopsy-related AEs

There were no FT drug-related AEs. There were no serious AEs (SAEs)

^a^Includes all randomized AS subjects. Crossover patient AEs included with AS group prior to crossover. Post-crossover AEs included in FT groups

^b^Self-limited and brief duration (*n* = 41/67 ≤ 4 days)

### Efficacy results (Table 3; Fig. 2): all comparisons unless otherwise listed are by Pearson exact chi-square

**Table 3:** Progression outcomes

	FT 2.5 mg	FT 15 mg	Pooled FT	AS
(a) % (Proportion) with Gleason ≥ 3 + 4, biopsies and RP surgical pathology				
18 months	16.7 (6/36)	8.8 (3/34)^1^	12.9 (9/70)^2^	41.2 (7/17)
36 months	26.9 (7/26)	18.2 (4/22)^3^	22.9 (11/48)^4^	56.3 (9/16)
48 months	62.5 (10/16)	33.3 (5/15)	48.4 (15/31)	71.4 (10/14)
(b) % (Proportion) with both Gleason ≥ 3 + 4 biopsies and RP surgical pathology, and interventions^a^				
18 months	9.8 (4/41)	7.7 (3/39)	8.8 (7/80)	36.8 (7/19)
36 months	16.7 (5/30)	14.3 (4/28)	15.5 (9/58)	47.1 (8/17)
48 months	30.4 (7/23)	16.7 (4/24)^5^	23.4 (11/47)^6^	62.5 (10/16)
(c) % (Proportion) with Gleason ≥ 3 + 4 biopsies and RP surgical pathology^b^				
18 months	12.8 (6/47)	6.5 (3/46)^7^	9.7 (9/93)^8^	25.9 (7/27)
36 months	18.9 (7/37)	11.8 (4/34)^9^	15.5 (11/71)^10^	34.6 (9/26)
48 months	37 (10/27)	18.5 (5/27)	27.8 (15/54)	41.7 (10/24)
(d) % (Proportion) with both Gleason ≥ 3 + 4 biopsies and RP surgical pathology, and interventions^a,b^				
18 months	7.7 (4/52)^11^	5.9 (3/51)^12^	6.8 (7/103)^13^	24.1 (7/29)
36 months	12.2 (5/41)	10 (4/40)^14^	11.1 (9/81)^15^	29.6 (8/27)
48 months	20.6 (7/34)	11.1 (4/36)^16^	15.7 (11/70)^17^	38.5 (10/26)

^a^RP, radiotherapy, or chemotherapy

^b^Including all patients with ≥ 2 biopsies

^1^*p* = 0.0102; ^2^*p* = 0.0129, ^3^*p* = 0.0199, ^4^*p* = 0.0265, ^5^*p* = 0.0059, ^6^*p* = 0.0064 (1–6 Pearson exact chi-square), ^7^*p* = 0.0199, ^8^*p* = 0.0288, ^9^*p* = 0.033, ^10^*p* = 0.0392, ^11^*p* = 0.0383, ^12^*p* = 0.0176, ^13^*p* = 0.0074, ^14^*p* = 0.0398, ^15^*p* = 0.0221, ^16^*p* = 0.011, ^17^*p* = 0.0166 (7–17 Pearson chi-square)

**Fig. 2:**
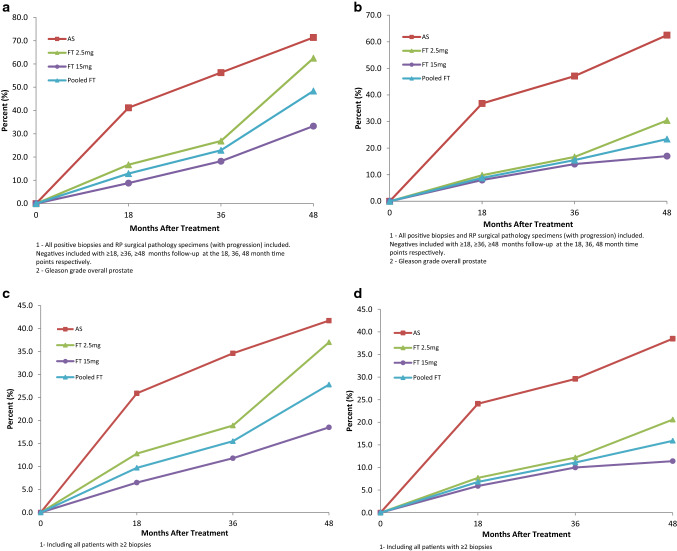
Outcomes of clinical and pathological progression vs time in FT-treated and AS control patients. a Cumulative percentage of patients with increased Gleason grade biopsies and/or RP surgical pathology specimens vs time after treatment. FT 15 mg and pooled FT groups statistically significant reduction vs AS control (in Table 3). b Cumulative percentage of patients with interventions (surgery and radiotherapy) with Gleason grade biopsies and/or RP surgical pathology specimens vs time after treatment. FT 15 mg and pooled FT groups’ statistically significant reduction vs SAS control (in Table 3). c Cumulative percentage of patients with increased Gleason grade vs time after treatment (as in a, also including all subjects with ≥ 2 biopsies). d Cumulative percentage of patients with increased Gleason grade and intervention vs time after treatment (as in b, also including all subjects with ≥ 2 biopsies).

At 18-month biopsies, primary endpoint was met for FT 15 mg (normal BLF in AS 36.8% vs FT 15 mg 71.1%, p = 0.0214; pooled FT 65.8%, NS; FT 2.5 mg 61%, NS). Secondary endpoints were as follows: (1) Median Gleason grade in each treatment group in BLF at 18 months was AS 3 + 3 (Group Grade 2 or higher in 4/19), vs benign for each of FT 15 mg (Group Grade 2 or higher in 1/38) (*p* = 0.0066), FT 2.5 mg (Group Grade 2 or higher in 2/41) (*p* = 0.0593), and pooled FT groups (Group Grade 2 or higher in 3/79) (*p* = 0.0109). (2) Median Gleason grade in each treatment group for entire prostate at 18 months was benign for FT 15 mg (Group Grade 2 or higher in 3/34) (*p* = 0.0044) and benign for pooled FT (Group Grade 2 or higher in 9/70) (*p* = 0.0086) vs 3 + 3 for AS (Group Grade 2 or higher in 7/17), and 3 + 3 for FT 2.5 mg (Group Grade 2 or higher in 6/36). (3) BLF mean tumor volume in each treatment group (estimated by biopsy %) at 18 months was pooled FT group (− 58.5%) vs AS controls (+ 68.8%, *p* = 0.0189, *t* test) (FT 15 mg, − 59%, FT 2.5 mg, − 58%). (4) Entire prostate mean tumor volume change at 18 months was significantly lower in pooled FT group (+ 41.5%) compared to AS controls (+ 279.7%, *p* = 0.0134) (FT 15 mg + 32.7%, FT 2.5 mg + 47.8%, *t* tests).

Data analysis of pathological progression (Table 3; Fig. 2a, b) was done with the exclusion of negative (ie non-progression) patients where there was no biopsy or surgical pathology data from the relevant time point or later. The pathological results for long-term Gleason grade (in addition to the median tumor grade progression differences above) included (all comparisons below by Pearson exact chi-square):

1. Percentage of subjects with increase of Gleason score to Grade Group 2 (Gleason score 3 + 4 = 7) or higher on follow-up biopsy or RP surgical pathology in prostate overall was significantly reduced in FT vs AS [18 months: AS 41.2% vs FT 15 mg 8.8% (− 78.6%, *p* = 0.0102); FT 2.5 mg 16.7% (− 59.5%, *p* = 0.0858)]; pooled FT 12.9% (− 68.7%, *p* = 0.0129); 36 months: AS 56.3% vs FT 15 mg 18.2% (− 67.7%, *p* = 0.0199); FT 2.5 mg 26.9%, (− 52.2% NS); pooled FT 22.9% (− 59.3%, *p* = 0.0265); 48 months: AS 71.4% vs FT 15 mg 33.3% (− 53.4%, *p* = 0.0656); FT 2.5 mg 62.5%, NS; pooled FT 48.4% (− 32.2%, NS). Median tumor grades at 36 months were Gleason 3 + 4 for AS vs Gleason 3 + 3 for each of FT 15 mg, FT 2.5 mg, pooled FT; and those at 48 months were Gleason 3 + 4 for AS vs 3 + 3 for FT 15 mg, pooled FT; and 3 + 4 for FT 2.5 mg.
2. Patients treated with FT 15 mg and pooled FT group had significantly less increase of treatment side Gleason score to Grade Group 2 (Gleason score 3 + 4 = 7) or higher at 18 and 36 months [18 months: AS 33.3% vs FT 15 mg 8.8% (− 73.6%, *p* = 0.0466); FT 2.5 mg 11.8% (− 64.6%, NS)]; pooled FT 10.3% (− 69.1%, *p* = 0.0365); 36 months: AS 50% vs FT 15 mg 18.2% (− 63.6%, *p* = 0.0665); FT 2.5 mg 20.8% (− 58.4%, NS); pooled FT 19.6% (− 60.8%, *p* = 0.0379).
3. Percentage of subjects with primary Gleason pattern (≥ 4) on follow-up biopsies and RP surgical pathology in prostate overall was reduced in pooled FT groups vs AS [18 months: AS 17.6% vs pooled FT 3.1% (− 82.4%, *p* = 0.0597)]; 36 months: AS 26.7% vs pooled FT 5.1% (− 80.9%, *p* = 0.0439). The percentage of AS subjects with biopsy primary pattern ≥ 4 at 3 years was 15.4%, compared to FT 2.5 mg 5%, FT 15 mg 5.2%, and pooled FT 5.1%.

Prospective assessment of PCa progression showed evidence of drug benefit. After 4 years, the percentage of control AS patients who received surgery or radiotherapy for their PCa was 68.4%, vs FT 15 mg 31% (− 54.7%, *p* = 0.0177), and vs combined FT groups 40% (− 41.5%, *p* = 0.0374). The percentage of control AS patients with surgery or radiotherapy with Gleason grade progression from baseline in the entire gland was 62.5%, vs FT 15 mg 16.7% (− 73.3%, *p* = 0.0059), vs FT 2.5 mg 30.4% (− 51.4%, *p* = 0.0586), and vs pooled FT dosages 23.4% (− 62.6%, *p* = 0.0064) (Fig. 2b, d). Total *n* of CO patients with pathological data ≥ 36 months: six (FT 15 mg/2.5 mg: 3 patients each) and ≥ 48 months: two (FT 15 mg/2.5 mg: 1 patient each); calculations without CO subjects had no significant effect on the above progression results. Two patients (both FT 2.5 mg), one with Gleason grade 5 + 3 (LTFU) and a second with Gleason 3 + 4 (site closed), were included in the pathological results, but were excluded from intervention results, because there was no confirmation of treatment despite efforts to follow-up. Two patients (FT 15 mg), one with Gleason grade 4 + 4, and a second with 4 + 3 (age 88), and one patient (FT 2.5 mg) with Gleason 3 + 4, were untreated after 5 years of surveillance, and were included in all results. Gleason upgrades overall were 67.9% in BLF or immediately adjacent quadrants, 32.1% were not adjacent, with 24.9% contralateral. Although the incidence of progression was significantly higher in untreated AS subjects, there were no significant differences between FT and AS groups in the above quadrant localization relative proportions. Consistency of quadrant localization reporting in SP specimens after study exit treatments had limitations due to non-uniform external processing and non-central reading after the 45-day biopsy.

Post hoc analysis of baseline PSA density showed a minority of patients (15.8%) had PSA density ≥ 0.15 ng/mL/cc^3^. There were no significant differences between treatment randomization groups re baseline proportions of higher PSA density patients, nor in proportions of patients with post-randomization Gleason grade increases in higher vs lower baseline PSA density patients. Post hoc sensitivity testing of Gleason grade progression was done by including all randomized subjects in all groups with ≥ 2 biopsies, which showed AS progression values of 25.9% and 34.6% at 18 and 36 months, compared to FT 15 mg 6.5% (*p* = 0.0199) and 11.8% (*p* = 0.033), and compared to pooled FT 9.7% (*p* = 0.0288) and 15.5% (*p* = 0.0392) (Table 3; Fig. 2c, d).

Dose–response long-term data showed FT 15-mg superiority to FT 2.5 mg in most endpoints and overall cancer progression. (1) Four-year incidence of surgery and radiotherapy for all causes was significantly reduced in the FT 15 mg group (31%), compared to control AS (68.4%, *p* = 0.0177), while the 2.5-mg group change (48.4%) was not statistically significant. (2) 3-year incidence of Gleason grade increase in prostate overall was significantly reduced (− 67.7%) in patients who received FT 15 mg (18.2%) compared to AS control group (56.3%, *p* = 0.0199) but in the FT 2.5 mg group (26.9%, *p* = 0.1009) was not. (3) Incidence of 18-month Gleason grade increase was significantly less in the FT 15-mg group compared to AS in the prostate overall (− 78.6%, *p* = 0.0102) and in the treated prostate lobe (− 73.6%, *p* = 0.0466), whereas the reductions in the 2.5-mg group did not reach statistical significance (− 59.5%, NS and − 64.6%, NS, respectively).

PSA mean change baseline to post-randomization nadir was significantly reduced in FT-treated groups but not in AS controls at 18 months (FT 2.5 mg − 16.7%, *p* = 0.0011; FT 15 mg − 18.6%, *p* = 0.0015); pooled FT groups − 17%, *p* < 0.0001 vs AS (−13.3%, NS) and at 4 years (FT 2.5 mg − 21.4%, *p* = 0.0004; FT 15 mg − 20.4%, *p* = 0.0065); pooled FT groups − 20.9%, *p* < 0.0001; AS (− 12.5%, NS) (one-sample *t* tests).

**Fig. 3:**
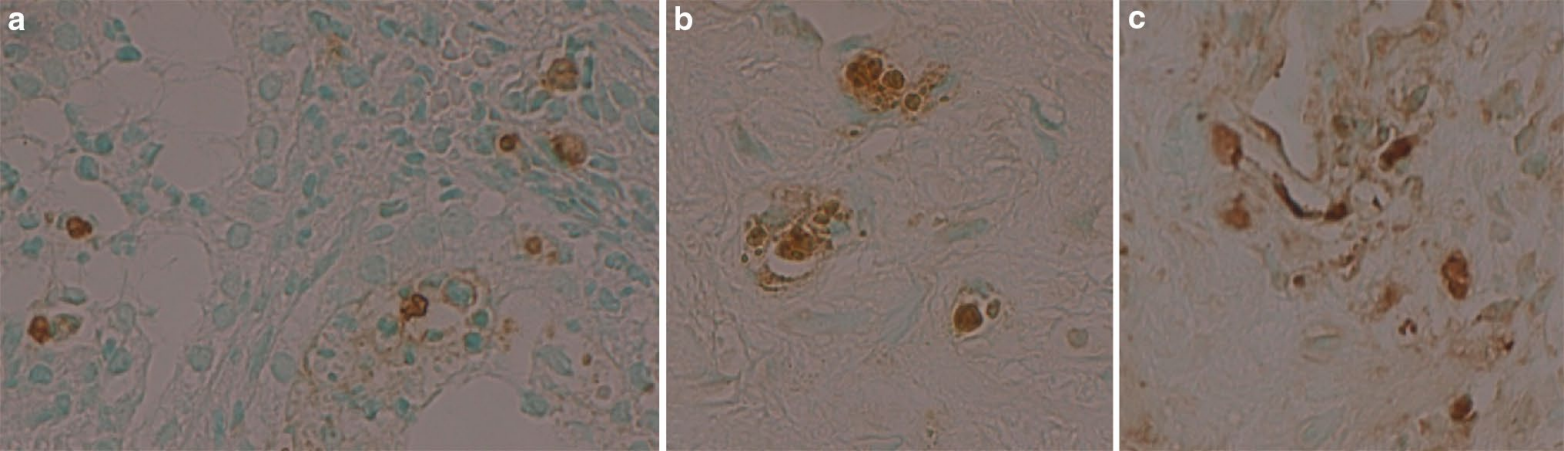
**a**–**c** Immunohistochemical TUNEL staining of 6-week biopsies from FT-treated patients showing TUNEL positivity (dark brown) indicating apoptosis of prostate glandular cells after FT injection into prostate. × 400 (figure courtesy of Nymox Corp)

With terminal deoxynucleotidyl transferase deoxyuridine triphosphate nick end (TUNEL) staining of biopsies, foci of glandular cells undergoing apoptotic cell death were identified in FT-treated subjects in their 45-day biopsies (Fig. 3). There was no TUNEL positivity identified in control AS biopsies. These histochemical results will be expanded in a separate research report.

## Discussion

The results of this long-term prospective study show that after a single targeted FT injection, there is evidence of statistically significant long-term inhibition of PCa progression both clinically and histologically. Clinical progression after 4 years was reduced by FT whether calculated by occurrence of interventions for PCa with increased Gleason grade in overall prostate (− 73.3% for FT 15-mg group compared to AS, − 62.6% for pooled FT groups); or by interventions with or without Gleason increase (− 54.7% for FT 15-mg group compared to AS; − 41.5% for pooled FT groups). Pathological progression after 3 years was diminished as determined by comparison of prostate overall incidence of increased Gleason grade (− 67.7% incidence of Gleason grade increase for FT 15-mg group compared to AS group, − 59.3% for pooled FT groups); and 3-year comparison of treatment side hemi-prostate (− 63.6% incidence of Gleason grade increase on side of FT 15-mg treatment compared to AS, − 60.8% for pooled FT groups). Three-year incidence of primary pattern ≥ 4 was reduced -80.9% in pooled FT patients compared to AS arm.

It should be emphasized that this study prospectively compared single injection of two different single doses of FT to an AS cohort. Better results may, therefore, be potentially possible after repeated injection(s). In BPH trials for FT, repeat injection has produced greater long-term improvement in BPH parameters compared to single-dose treatment, without any additional safety risk [37, 38, 41–43].

The injections in this study were targeted by routine ultrasound to the quadrant where the qualifying biopsy had identified the baseline T1c PCa single lesion. Although FT is not measurable (not present) by pharmacokinetic sampling outside of prostate at any time point after injection [37–40], the injection of 10-mL FT solution can be visualized by ultrasound to diffuse within the hemi-prostate well beyond the 10-mL volume space [41–43]. Statistically significant reduced PCa progression within the treatment side hemi-prostate was unexpected, but can probably in part be explained by the visualized diffusion of FT within the ipsilateral lobe and contact with other foci of undetected tumor at the time of treatment. Enhanced imaging is expected to improve targeting accuracy compared to TRUS alone. However for the reasons cited above, the 10-mL injection with visualized diffusion by TRUS was likely to have reached much more of the prostate lobe than only the targeted lesion.

It is unknown at this time if the beneficial effect in outcomes with FT in this study is due to destruction of primary grade 6 foci some of which would have progressed to higher grade, or due to destruction of pre-existent undetected higher grade foci different from the baseline focus identified, or to missed higher grade foci within the initial focus, or whether the decrease in progression compared to AS is through another hypothetical mechanism such as inhibition of other premalignant cellular targets, or combinations of two or more of the above. Discussion of these currently unverifiable mechanisms is beyond the scope of this report. The higher Gleason grade foci found in the patients with progression were 67.9% in BLF or adjacent quadrants, and only 24.9% were contralateral, which suggests that most progression was emanating (whether de novo or pre-existent) from the baseline identified foci or closely adjacent tissue.

The main potential roles of a validated non-chemotherapeutic non-toxic molecular approach to low-grade PCa are (1) the possibility to delay treatment interventions which may be associated with undesirable secondary effects, and (2) the option to continue an ongoing AS strategy yet offer patients an opportunity for potential cancer ablation. For many patients, this adjunct to surveillance might be helpful for the persistent and considerable uncertainties, anxieties, and psychological/emotional burdens, which may negatively impact quality of life when only selecting AS.

There are limitations to this long-term study: (1) single dosing was utilized and the additional potential benefits of multiple dosing were not explored in cohorts, (2) potential combination(s) with other non-interventional modalities (e.g., molecular) was not addressed, (3) patients with more extensive low-grade PCa were not in the trial, so the benefit for these important groups remains unknown (e.g., Gleason 6 with biopsy proven multifocal disease, Gleason 6 with lesion(s) > 50% of biopsy core, Gleason 3 + 4, and others), (4) prospective sub-groups were not explored (e.g., ethnic; and phenotypic sub-groups), (5) MRI, which would help to define intervention sites, was not a required part of the 2012 protocol for this study, and (6) blinded central review was done on all first-year biopsies (*n* = 287), but subsequent biopsies and surgical pathology from RP specimens were done at the local institutions where standardization was less uniform and inter-observer variability was a potential source of imprecision. All of these additional aspects, and perhaps, other considerations remain to be addressed in further studies.

This study was a prospective randomized multi-center parallel group controlled study designed to test safety and effectiveness of FT treatment and AS compared to AS alone, in a group of 147 men with recent biopsy Grade Group 1 PCa. Larger single-center studies of AS cohorts have reported histological progression rates in the 12–51% range after median times of 1.5–6.4 years, most commonly in the 25–35% range largely depending upon criteria and methodology [44–55], and these percentages are lower than the calculated values for the AS control group in the present study. The former are based on AS protocols (which have evolved over time) and the published rates have varied depending on whether there was repeat biopsy at baseline (repeat biopsies may exclude subjects with higher baseline Gleason missed on initial biopsy); frequency of biopsies; baseline population characteristics; extent of sampling, and other factors [44–49]. The present study was designed to test treatment effect and (1) did not entail a repeat biopsy prior to enrollment, (2) there were higher core numbers per biopsy (16-core compared to usually 8–12 in the large series), (3) there were more frequent biopsies (5 biopsies by 5-year time compared to the more usual ≥ 2 biopsies in the AS protocols, (4) the study had smaller *n* (not comparable to major AS studies), and (5) included RP surgical pathology results in all patients where this was available after randomization. All of the preceding will give the AS group (and equally so the drug groups in this study) potentially higher (more conservative) numbers of grade progression results [49–55]. Furthermore, all three groups (AS, FT 2.5 mg and 15 mg) had significant numbers of subjects (e.g,. 33% of AS group) with two biopsies which were negative but who were excluded from analysis due to early patient withdrawal, and would have qualified as negative under a different criterion of ≥ 2 biopsies negative. Progression to Gleason ≥ 4 + 3 in the AS group in this study (15.4% at 3 years, biopsy data; 8.7% if all subjects with ≥ 2 biopsies are included) was not appreciably higher than the published literature [44–49] which supports that the AS group was representative. A post hoc sensitivity test showed AS progression values in this study are comparable to published AS values if all randomized subjects in all groups with ≥ 2 biopsies are included (Table 3c, d): AS 25.9% and 34.6% at 18 and 36 months, compared to FT 15 mg 6.5% (*p* = 0.0199) and 11.8% (*p* = 0.033), and compared to pooled FT 9.7% (*p* = 0.0288) and 15.5% (*p* = 0.0392). PCa progression under AS is complex and depends on many factors (e.g., PSA density, extent of baseline cancer, imaging status, schedule and extent of surveillance, and other factors still being clarified), and even when there are attempts to control these factors, there is still some degree of discordance among different published single-center cohorts [49].

PCa is the second most common cancer in men (after skin cancer), and is the most common internal cancer in men. With modern era urological diagnostics and therapies, the election for AS approaches has increased. The process of repeated biopsies, examinations, and PSA testing increases patients' anxieties, and there are significant discomforts and potential side effects with the monitoring process (such as biopsy-related urosepsis). Additionally, when conducting AS for Grade Group 1 patients, there does exist the potential for progression of histopathology. Balancing the risks of surgical or radiation interventions vs the risks and anxieties of monitoring and surveillance is a cause of stress and uncertainty for some patients. After 3 years in the reported study (including all patients with ≥ 2 biopsies and including RP surgical pathology results), 29.6% of AS patients had interventions with Gleason grade progression > 3 + 3, and 34.6% of patients had Gleason grade > 3 + 3. Regardless of whether the subjects were prospectively or retrospectively categorized as low risk or very low risk, all were under AS in the protocol. There were no recorded deaths from PCa in any treated or untreated subjects after 78 months in the trial, which is consistent with the low risk of Grade Group 1 PCa.

Urologists have long recognized the unmet need for prostate cancer treatments that can contribute to improved outcomes for their patients together with reduced side effects and stresses that may significantly impact on quality of life. The goal of a therapy such as FT injectable is to allow for an initial and less toxic treatment for low-risk prostate cancer patients, achieving the benefits of molecular ablation with minimal risk of side effects.
